# Conformational Characterization of Native and L17A/F19A-Substituted Dutch-Type β-Amyloid Peptides

**DOI:** 10.3390/ijms21072571

**Published:** 2020-04-07

**Authors:** Kai-Cyuan He, Yi-Ru Chen, Chu-Ting Liang, Shi-Jie Huang, Chung-Ying Tzeng, Chi-Fon Chang, Shing-Jong Huang, Hsien-Bin Huang, Ta-Hsien Lin

**Affiliations:** 1 Basic Research Division, Medical Research Department, Taipei Veterans General Hospital, Taipei 11217, Taiwan; kay5402@hotmail.com; 2 Institute of Biochemistry & Molecular Biology, National Yang-Ming University, Taipei 11221, Taiwan; ja7yrx326159487@gmail.com; 3 Department and Institute of Pharmacology, National Yang-Ming University, Taipei 11221, Taiwan; YRC_Chen@adimmune.com.tw; 4 Department of Life Sciences and Institute of Genome Sciences, National Yang-Ming University, Taipei 11221, Taiwan; ayumi850@gmail.com (C.-T.L.); wetlandyuhu@gmail.com (C.-Y.T.); 5 Genomics Research Center, Academia Sinica, Taipei 11529, Taiwan; chifon@gate.sinica.edu.tw; 6 Instrumentation Center, National Taiwan University, Taipei 10617, Taiwan; shingjonghuang@ntu.edu.tw; 7 Department of Life Science and the Institute of Molecular Biology, National Chung Cheng University, Chia-yi 62102, Taiwan

**Keywords:** NMR, CD, Aβ, β-amyloid peptide, α/β-discordant, Dutch-type mutation, E22Q, familial Alzheimer’s disease, FAD

## Abstract

Some mutations which occur in the α/β-discordant region (resides 15 to 23) of β-amyloid peptide (Aβ) lead to familial Alzheimer’s disease (FAD). In vitro studies have shown that these genetic mutations could accelerate Aβ aggregation. We recently showed that mutations in this region could alter the structural propensity, resulting in a different aggregative propensity of Aβ. Whether these genetic mutations display similar effects remains largely unknown. Here, we characterized the structural propensity and aggregation kinetics of Dutch-type Aβ_40_ (Aβ_40_(E22Q)) and its L17A/F19A-substituted mutant (Aβ_40_(L17A/F19A/E22Q)) using circular dichroism spectroscopy, nuclear magnetic spectroscopy, and thioflavin T fluorescence assay. In comparison with wild-type Aβ_40_, we found that Dutch-type mutation, unlike Artic-type mutation (E22G), does not reduce the α-helical propensity of the α/β-discordant region in sodium dodecyl sulfate micellar solution. Moreover, we found that Aβ_40_(L17A/F19A/E22Q) displays a higher α-helical propensity of the α/β-discordant region and a slower aggregation rate than Aβ_40_(E22Q), suggesting that the inhibition of aggregation might be via increasing the α-helical propensity of the α/β-discordant region, similar to that observed in wild-type and Artic-type Aβ_40_. Taken together, Dutch-type and Artic-type mutations adopt different mechanisms to promote Aβ aggregation, however, the L17A/F19A mutation could increase the α-helical propensities of both Dutch-type and Artic-type Aβ_40_ and inhibit their aggregation.

## 1. Introduction

On the basis of the amyloid cascade hypothesis [1,2], aggregation of β-amyloid peptide (Aβ) is a crucial factor for the neuronal damage that leads to Alzheimer’s disease (AD). The clinical hallmarks of AD are neurofibrillary tangles and senile plaques within AD patients’ brains. The major components of these two hallmarks are tau protein and Aβ, respectively. Aβ, about 38–42 residues in length, is a derivative from sequentially enzymatic processing of transmembrane protein, called β-amyloid precursor protein (βAPP). It has been reported that increased Aβ production resulting from mutations in the processing enzymes of βAPP (such as β- and γ-secretase) [3] or βAPP mutations close to the cutting site of the processing enzymes [4,5] would cause family Alzheimer’s disease (FAD). Point mutations within the Aβ region of βAPP have also been shown to cause family Alzheimer’s disease (FAD), such as mutations occurring at A21 [6], E22 [7,8], and D23 [9,10] of Aβ. Several studies have shown that E22G (Arctic-type mutation), E22Q (Dutch-type mutation), and D23N (Iowa-type mutation) mutations would alter the aggregation behavior [11] and structure property [12,13,14,15,16,17] of Aβ.

Structures of wild-type Aβ in different environments have been reported. They adopted a mainly random coil conformation [18] or a short α-helical structure in aqueous solution [19]. In SDS micellar solution, two short α-helices were contained [20,21,22]. In the presence of large unilamellar vesicles (zwitterionic lipid bilayers), a partially folded structure was shown [23]. In vitro experiments have shown that Aβ would aggregate into fibrils whose secondary structure was mainly β-sheets [24,25,26]. Similar β-sheet conformations were also observed for the Aβ fibrils purified from AD brain tissue [27,28]. These findings suggested that Aβ would undergo conformational transitions from random coil or α-helix conformation into β-sheet structure during the process of aggregation. However, the detailed mechanism of the aggregation process of Aβ remains unclear. The aggregative propensity of Aβ is linked to its structural conversion tendency which depends on its intrinsic structural propensity and the local environments where it exists.

Previously, we reported that mutations located in the α/β-discordant region (resides 15 to 23) of Aβ (E22G and L17A/F19A mutations) could either reduce or augment α-helical propensity of Aβ, leading to either an increase or a decrease of the rates of structural transition and fibril formation of Aβ [21,29,30,31]. The results of these studies support the view that the α-helical and aggregative propensities of Aβ tend to be inversely correlated. It remains uncertain whether other FAD-related mutations located in the Aβ sequence would promote Aβ aggregation by reducing the α-helical propensity of Aβ or not. We have been focusing on investigating the effects of FAD-related mutations in the α/β-discordant region of Aβ on the structural propensity of Aβ. The effect of Arctic-type mutation (E22G) on the structural propensity of Aβ has been reported [16,30], however, the effects of other FAD-related mutations on the structural propensity of Aβ remain largely unknown. In the present study, we applied nuclear magnetic resonance (NMR) and circular dichroism (CD) spectroscopies to characterize the structural conformation of Dutch-type Aβ_40_ (Aβ_40_(E22Q) in SDS micellar solution. Moreover, the effects of Ala replacements at L17 and F19, which have been shown to increase the α-helical propensity and decrease the rate of aggregation of wild-type Aβ_40_ and Arctic-type Aβ_40_ (Aβ_40_(E22G)), on the structure and aggregation kinetics of Dutch-type Aβ_40,_ were also characterized. Our data suggested that the structural conformation of Aβ_40_(E22Q) in SDS micellar solution is very similar to that of wild-type Aβ_40_. There is only a slight difference between these two structures. However, there is a more significant difference in α-helical propensity between Aβ_40_(E22Q) and Aβ_40_(L17A/F19A/E22Q). These results are discussed in terms of the relation between the structural and aggregative propensities of Aβ mutants.

## 2. Results

### 2.1. Comparison of the Secondary Structures of Wild-Type Aβ_40_ and Aβ_40_(E22Q)

**Figure 1:**
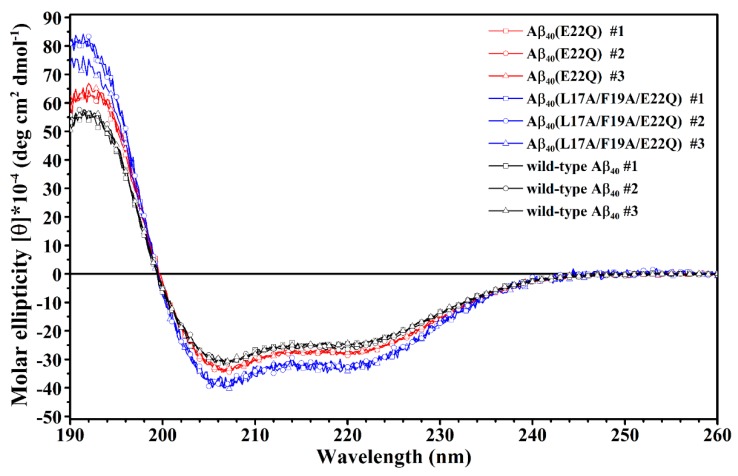
Overlay of CD spectra of wild-type Aβ_40_ (**black**), Aβ_40_(E22Q) (**red**) and Aβ_40_(L17A/F19A/E22Q) (**blue**) in 100 mM SDS micellar solution.

In our recent study, we reported the effect of Arctic-type mutation (E22G) on the structure of Aβ in SDS micellar solution. To gain more insight into the effect of FAD-related mutation at position 22 on the structure of Aβ, we characterized the structure of Aβ_40_(E22Q) in the present study. Aβ_40_(E22Q) had been found in FAD patients with severe cerebral amyloid angiopathy (CAA). To examine the effect of E22Q mutation on the structure of Aβ, we first analyzed the secondary structures of wild-type Aβ_40_ and Aβ_40_(E22Q) in SDS micellar solution using circular dichroism (CD) spectroscopy. It can be seen from Figure 1 that the CD spectrum of wild-type Aβ_40_ shows a band with positive ellipticity at around 192 nm and two bands with negative ellipticity at 207 nm and 221 nm which are CD spectral characteristics of α-helix, suggesting that the secondary structure content of wild-type Aβ_40_ in micellar solution is mainly α-helix. The result is consistent with that obtained in the previous studies [30,31]. The CD spectrum of Aβ_40_(E22Q) displays a similar spectral pattern to that of wild-type Aβ_40_ with a more positive ellipticity at around 192 nm and a slightly more negative ellipticity at 207 nm and at 221 nm, suggesting that Aβ_40_(E22Q) adopts mainly α-helical conformation as well, and the α-helix content of Aβ_40_(E22Q) might be slightly higher than that of wild-type Aβ_40_.

**Figure 2:**
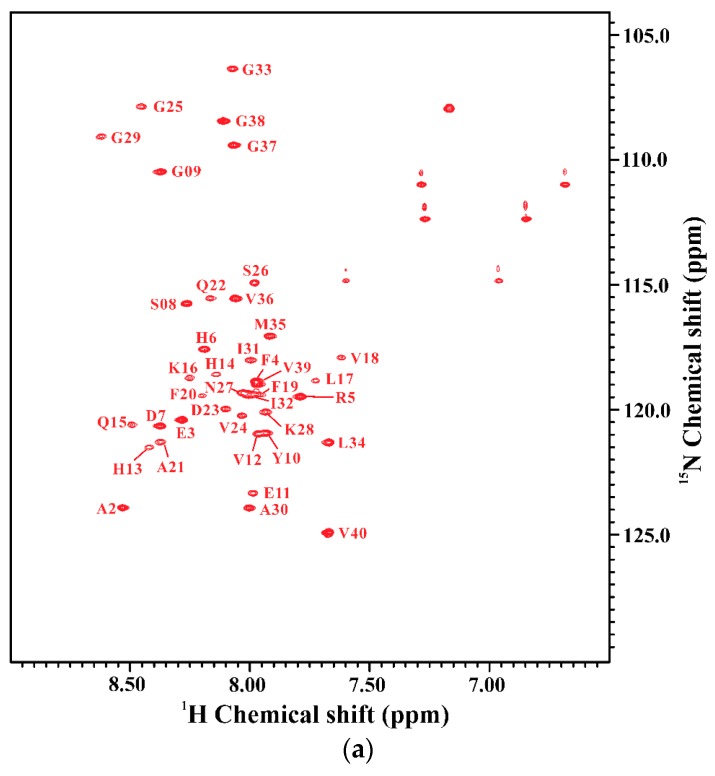

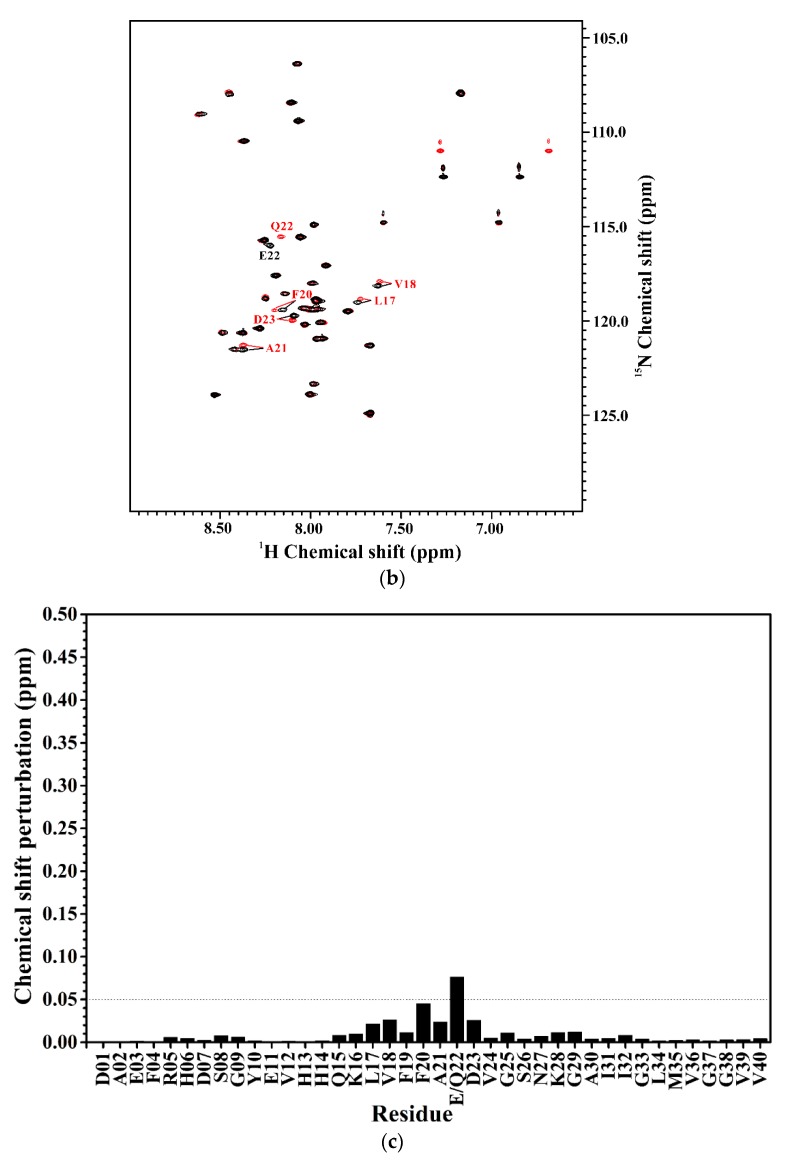
(**a**) Two-dimensional ^1^H-^15^N-HSQC spectrum of ^15^N-labeled Aβ_40_(E22Q) in 100 mM SDS micellar solution at 296 K; (**b**) Overlay of two-dimensional ^1^H-^15^N-HSQC spectra of ^15^N-labeled wild-type Aβ_40_ (black) and Aβ_40_(E22Q) (red) in 100 mM SDS micellar solution at 296 K. Residues which display chemical shift perturbations were labeled; (**c**) Chemical shift perturbation plotted as a function of residue number. Chemical shift perturbation was calculated using the equation [(^HN^Δppm)^2^ + (NΔppm/10)^2^]^1/2^, where ^HN^Δppm and ^N^Δppm were equal to ^1^H^N^ and ^15^N chemical shift differences between wild-type Aβ_40_ and Aβ_40_(E22Q), respectively [31].

We further applied NMR spectroscopy to characterize the secondary structure of Aβ_40_(E22Q) in SDS micellar solution. In order to derive the secondary structure from the backbone atom chemical shifts, we first accomplished the sequential backbone assignment of Aβ_40_(E22Q). Figure 2A shows the two-dimensional ^1^H-^15^N-HSQC spectrum of ^15^N-labeled Aβ_40_(E22Q) in SDS micellar solution. The result of residue assignment is shown in the figure. By comparison of the two-dimensional ^1^H-^15^N-HSQC spectrum of Aβ_40_(E22Q) with that of wild-type Aβ_40_, we obtained the effect of E22Q mutation on the two-dimensional ^1^H-^15^N-HSQC spectrum of wild-type Aβ_40_. Figure 2B showed the superimposed two-dimensional ^1^H-^15^N-HSQC spectra of wild-type and Dutch-type Aβ_40_. It is evident that these two spectra look almost the same except for some amide proton and nitrogen cross-peaks which displayed chemical shift changes as a result of E22Q mutation. According to the previously assigned two-dimensional ^1^H-^15^N-HSQC spectrum of wild-type Aβ_40_ [30], some cross-peaks which displayed relatively significant chemical shift changes on account of E22Q mutation were readily assigned to L17, V18, F20, A21, and D23 (excluding E22). In general, there are three major factors which contribute to the observed chemical shift perturbations of nitrogen (^15^N) and amide proton (^1^HN), including the sequence effect caused by E22Q mutation, the conformational change induced by E22Q mutation, and the interaction with SDS micelle altered by E22Q mutation. Further analysis revealed that the chemical shift perturbations are very small (less than 0.05) as shown in Figure 2C, suggesting that the effects of E22Q mutation on these three factors which cause chemical shift perturbations are very small. It can also be seen from Figure 2C that residues which displayed relatively significant chemical shift perturbations resulting from E22Q mutation were located in the α/β-discordant region (resides 15 to 23). This observation suggested that E22Q mutation might slightly affect the structural conformation of the α/β-discordant region of Aβ and/or the interaction of the α/β-discordant region of Aβ with SDS micelle.

**Figure 3:**
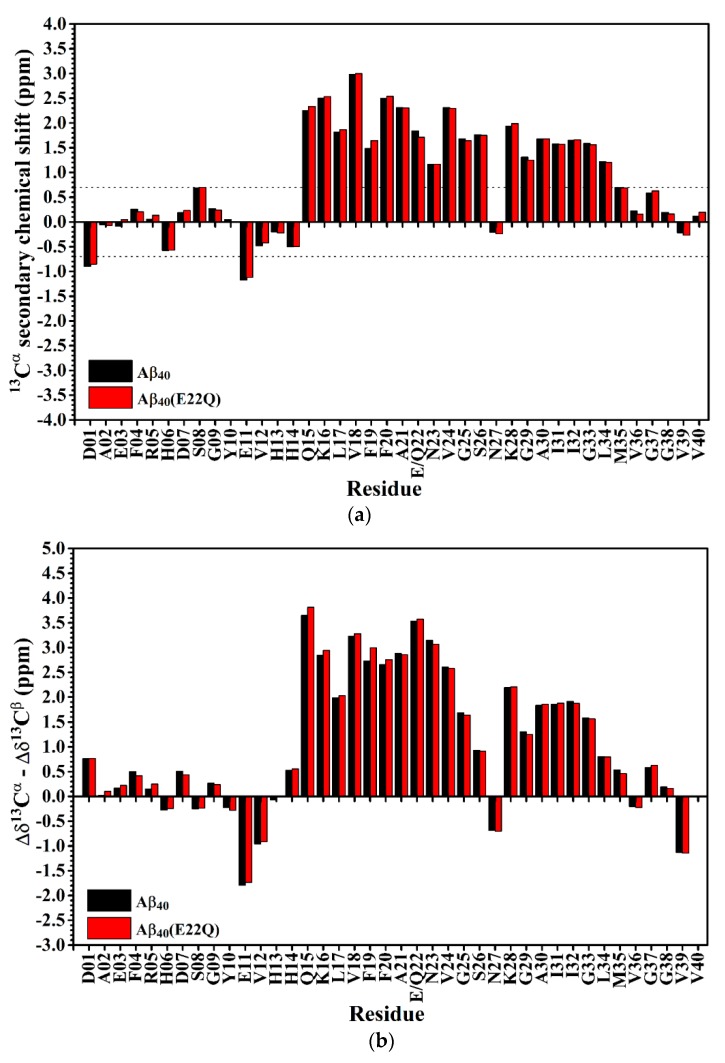
(**a**) ^13^C^α^ secondary chemical shifts of wild-type Aβ_40_ (balck) and Aβ_40_(E22Q) (red) plotted as a function of residue. In principle, if the ^13^C^α^ secondary chemical shift of an amino acid residue is greater than 0.7 ppm, its conformation would be α-helical [35]; (**b**) Differences between Δδ^13^C^α^ (^13^C^α^ secondary chemical shift) and Δδ^13^C^β^ (^13^C^β^ secondary chemical shift) of wild-type Aβ_40_ (black) and Aβ_40_(E22Q) (red) plotted as a function of residue. Δδ^13^C^α^ (or Δδ^13^C^β^) was defined as the difference between the observed ^13^C^α^ (or ^13^C^β^) chemical shift of an amino acid residue and its ^13^Cα (or ^13^Cβ) chemical shift in a random coil conformation. If Δδ^13^C^α^–Δδ^13^C^β^ for an amino acid residue is positive, its conformation would be α-helical. For a more detailed description of the relationship between the value of Δδ^13^C^α^–Δδ^13^C^β^ and secondary structure of an amino acid residue please see the reference [34].

In order to confirm the inference that the effect of E22Q mutation on the structural conformation is small, we used secondary chemical shifts of ^13^C^α^ and ^13^C^β^ which are mainly affected by the backbone conformation of the amino acid itself instead of any direct through-space interaction, slightly affected by the sequence [32], to estimate the secondary structure of Aβ_40_(E22Q) [33,34,35]. Figure 3A shows the comparison of ^13^C^α^ secondary chemical shifts of wild-type Aβ_40_ and Aβ_40_(E22Q). It is apparent that the ^13^C^α^ secondary chemical shifts of wild-type Aβ_40_ and Aβ_40_(E22Q) look almost the same except for a few residues in the α/β-discordant region which displayed slightly more positive ^13^C^α^ secondary chemical shifts resulting from E22Q mutation. This result suggested that both wild-type Aβ_40_ and Aβ_40_(E22Q) adopted two short α-helices from residues 15 to 26 and residues 28 to 34 [35] and a few residues in the α/β-discordant region might have adopted slightly higher α-helical propensities (α-helicity) [33] as a result of E22Q mutation. By taking the ^13^C^β^ secondary chemical shift into account, we further analyzed the effect of the E22Q mutation on the secondary structure of wild-type Aβ_40_. The results are shown in Figure 3B. As expected, the differences between ^13^C^α^ and ^13^C^β^ secondary chemical shifts of wild-type Aβ_40_ and Aβ_40_(E22Q) look almost the same. It can be seen from Figure 3B that a few residues in the α/β-discordant region also displayed slightly more positive values of Δδ^13^C^α^ –Δδ^13^C^β^ for Aβ_40_(E22Q) than for wild-type Aβ_40_. This observation suggested that the E22Q mutation might result in a slight increase in the α-helical propensities of a few residues in the α/β-discordant region as well [34]. These findings were consistent with those observed from CD spectroscopy. Since the slight differences in ^13^C^α^ secondary chemical shifts (or Δδ^13^C^α^–Δδ^13^C^β^) between wild-type Aβ_40_ and Aβ_40_(E22Q) are within the error limits of chemical shift measurements using three-dimensional NMR spectra, one may argue that these relatively small differences might be overinterpreted. These differences might merely come from sequence effect. At any rate, we may speculate that the effects of E22Q mutation on the secondary structure of Aβ and the interaction of Aβ with SDS micelle are insignificant. Even though it exists, it is very small according to our NMR and CD data.

### 2.2. Comparison of the Secondary Structures of Aβ_40_(E22Q) and Aβ_40_(L17A/F19A/E22Q)

**Figure 4:**
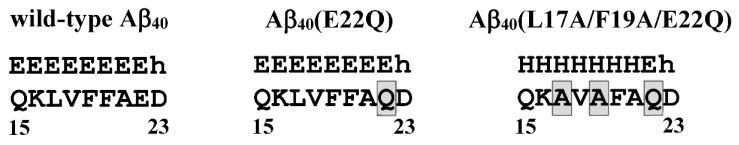
The predicted secondary structures of the α/β-discordant regions of wild-type Aβ_40_ Aβ_40_(E22Q) and Aβ_40_(L17A/F19A/E22Q). β-strands predicted with high and low probability were denoted by the symbols E and e, respectively. α-helical structures predicted with high and low probability were denoted by the symbols H and h, respectively [31,36].

In our recent study, we showed that residues L17 and F19 of Aβ played an important role in the structural and aggregative propensities of wild-type Aβ_40_ and Aβ_40_(E22G) [21,29,31]. To examine whether the effects of Ala replacements at L17 and F19 on the structure and aggregative property of Aβ_40_(E22Q) are similar to those observed for wild-type Aβ_40_ and Aβ_40_(E22G) or not, we performed structural characterization and aggregation kinetic study on Aβ_40_(L17A/F19A/E22Q). Prior to the experimental structural characterization of Aβ_40_(L17A/F19A/E22Q), we applied propensity-based prediction to the analyzed effects of E22Q and L17A/F19A mutations on the structural propensity of the α/β-discordant region of wild-type Aβ_40_ and Aβ_40_(E22Q), respectively [31,36]. The results obtained from in silico studies implied that wild-type Aβ_40_ and Aβ_40_(E22Q) adopt the same structural propensity in their α/β-discordant region. Unlike the E22G mutation which would alter the structural propensity of D23 from α-helix to β-strand, the E22Q mutation has no effect on the structural propensity of wild-type Aβ_40_. It can also be seen that the L17A/F19A mutation would alter the structural propensities of residues 15 to 21 in the α/β-discordant region of Aβ_40_(E22Q) from β-strand to α-helix as shown in Figure 4. The same effect has also been observed on wild-type Aβ_40_ and Aβ_40_(E22G) [31].

We next applied CD spectroscopy to examine the effect of L17A/F19A mutation on the overall secondary structure of Dutch-type Aβ_40_. The CD spectra of Aβ_40_(L17A/F19A/E22Q) are shown in Figure 1. It is apparent that Aβ_40_(E22Q) and Aβ_40_(L17A/F19A/E22Q) exhibited similar spectral patterns in their CD spectra, suggesting that the overall secondary structure of Aβ_40_(L17A/F19A/E22Q) is similar to that of Aβ_40_(E22Q). They both adopt mainly α-helical structures in SDS micellar solution. However, it can be seen from Figure 1 that Aβ_40_(L17A/F19A/E22Q) displayed more positive ellipticity at around 192 nm and more negative ellipticity at 207 nm and 221 nm than Aβ_40_(E22Q), suggesting that the L17A/F19A mutation would result in an increase of the α-helix content of Aβ_40_(E22Q). The difference between the CD spectra of Aβ_40_(E22Q) and Aβ_40_(L17A/F19A/E22Q) is more significant than that between wild-type Aβ_40_ and Aβ_40_(E22Q), indicating that the effect of L17A/F19A mutation on the overall secondary structure of Dutch-type Aβ_40_ is more prominent than that of E22Q mutation on the overall secondary structure of wild-type Aβ_40_.

**Figure 5:**
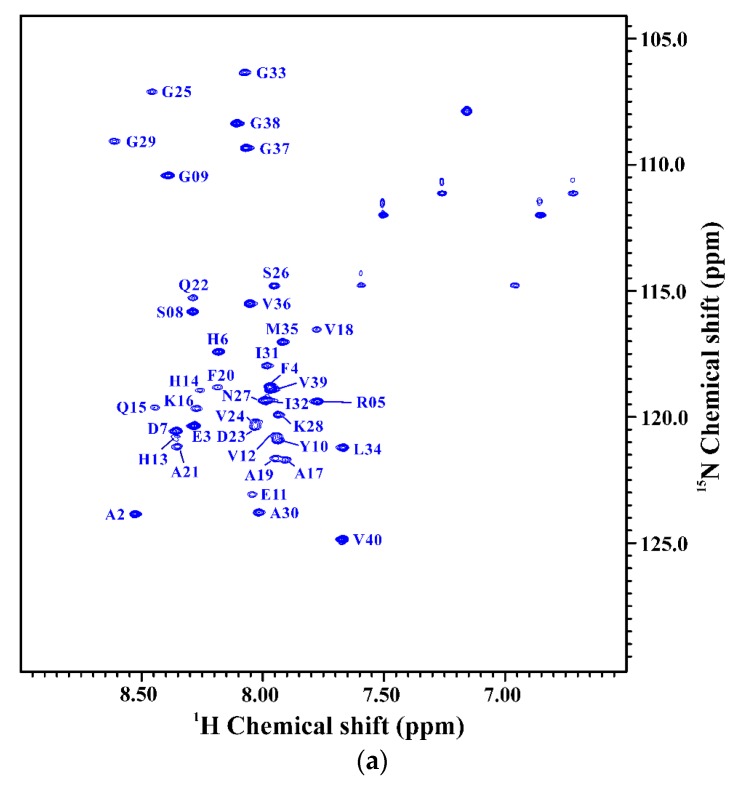

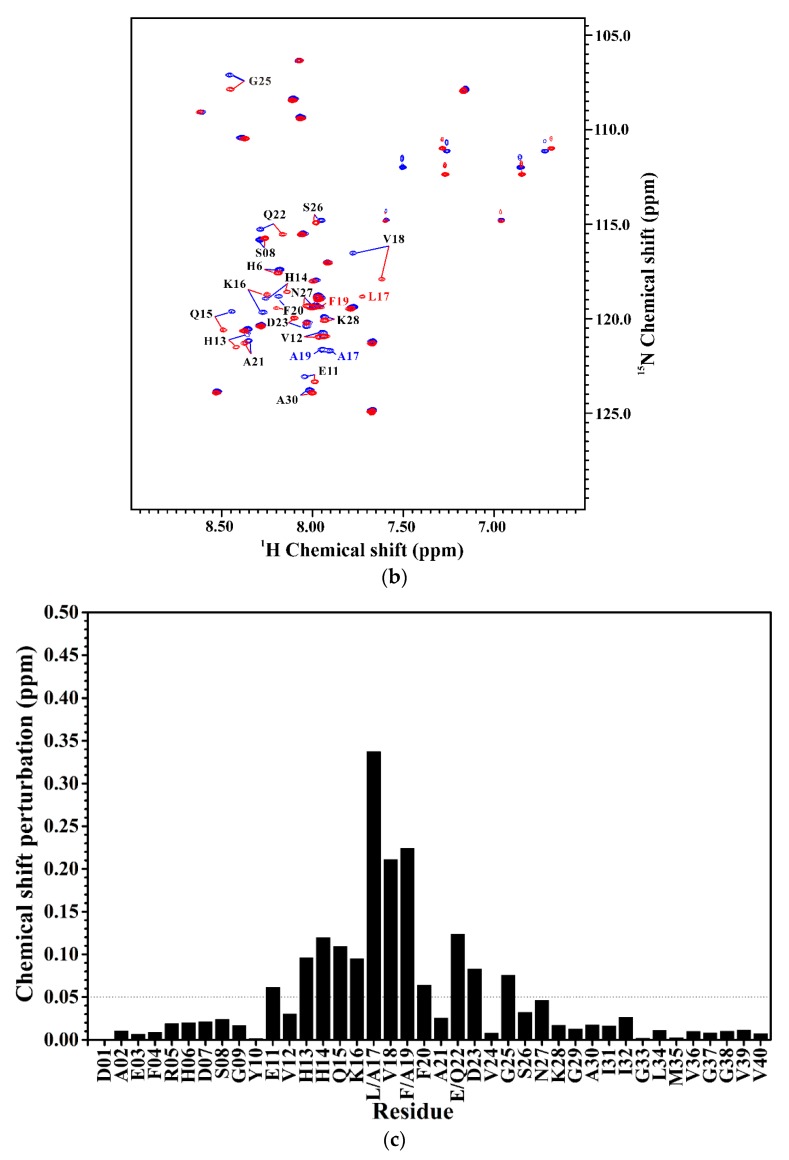
(**a**) Two-dimensional ^1^H-^15^N-HSQC spectrum of ^15^N-labeled Aβ_40_(L17A/F19A/E22Q) in 100 mM SDS micellar solution at 296 K; (**b**) Overlay of Two-dimensional ^1^H-^15^N-HSQC spectra of ^15^N-labeled Aβ_40_(E22Q) (red) and Aβ_40_(L17A/F19A/E22Q) (blue) in 100 mM SDS micellar solution at 296 K. Residues which display chemical shift perturbations were labeled; (**c**) Chemical shift perturbation plotted as a function of residue number. Chemical shift perturbation was calculated using the equation [(^HN^Δppm)^2^ + (^N^Δppm/10)^2^]^1/2^, where ^HN^Δppm and ^N^Δppm were equal to ^1^HN and ^15^N chemical shift differences between Aβ_40_(E22Q) and Aβ_40_(L17A/F19A/E22Q), respectively [31].

We also applied NMR spectroscopy to characterize the secondary structure of Aβ_40_(L17A/F19A/E22Q) in SDS micellar solution and used the same approach as that employed for analyzing the effect of E22Q mutation on the structural conformation of Aβ to analyze the effect of L17A/F19A mutation on the structural conformation of Dutch-type Aβ_40_. A two-dimensional ^1^H-^15^N-HSQC spectrum of ^15^N-labeled Aβ_40_(L17A/F19A/E22Q) in SDS micellar solution with the result of residue assignment is shown in Figure 5A. Figure 5B shows the comparison of the two-dimensional ^1^H-^15^N-HSQC spectra of Aβ_40_(E22Q) and Aβ_40_(L17A/F19A/E22Q). It is quite obvious that many amide proton and nitrogen cross-peaks of Aβ_40_(E22Q) display significant chemical shift changes because of L17A/F19A mutation. Cross-peaks which display significant chemical shift changes are indicated in the figure. Calculations of chemical shift perturbations were also performed for further analysis of the effect of the L17A/F19A mutation on the chemical shifts of the amide proton and nitrogen cross-peaks of Aβ_40_(E22Q). The results are shown in Figure 5C. Residues which exhibited significant chemical shift perturbations (greater than 0.05) were readily identified as E11, H13-F20 (excluding L17 and F19), Q22, D23, and G25. These residues are mainly located in the α/β-discordant region of Aβ_40_(E22Q), suggesting that the increases of α-helical content observed from CD spectra are mainly from the residues in the α/β-discordant region of Aβ_40_(L17A/F19A/E22Q). Similar effects have also been observed on wild-type Aβ_40_ and Aβ_40_(E22G) [31]. This finding implied that the L17A/F19A mutation would affect the structural conformation of the α/β-discordant region of Aβ_40_(E22Q) and the interaction of the α/β-discordant region of Aβ_40_(E22Q) with SDS micelle.

**Figure 6:**
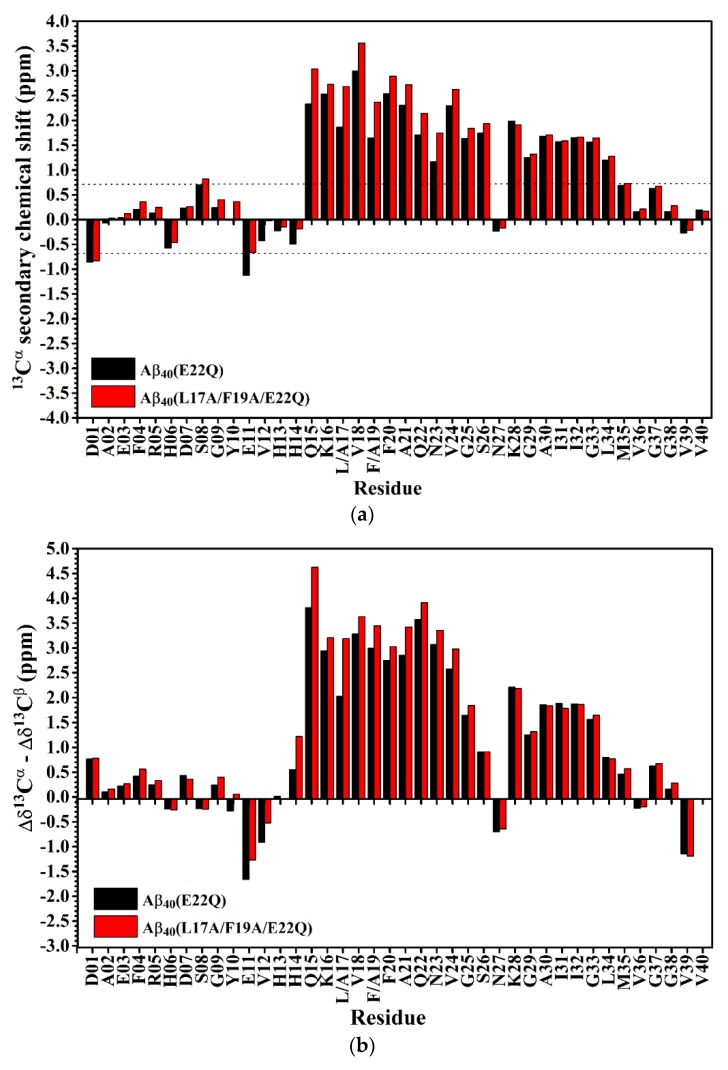
(**a**) ^13^Cα secondary chemical shifts of Aβ_40_(E22Q) (black) and Aβ_40_(L17A/F19A/E22Q) (red) plotted as a function of residue. In principle, if the ^13^C^α^ secondary chemical shift of an amino acid residue is greater than 0.7 ppm, its conformation would be α-helical [35]; (**b**) Differences between Δδ^13^C^α^ (^13^C^α^ secondary chemical shift) and Δδ^13^C^β^ (^13^C^β^ secondary chemical shift) of Aβ_40_(E22Q) (black) and Aβ_40_(L17A/F19A/E22Q) (red) plotted as a function of residue. Δδ^13^C^α^ (or Δδ^13^C^β^) was defined as the difference between the observed ^13^C^α^ (or ^13^C^β^) chemical shift of an amino acid residue and its ^13^C^α^ (or ^13^C^β^) chemical shift in a random coil conformation. If Δδ^13^C^α^–Δδ^13^C^β^ for an amino acid residue is positive, its conformation would be α-helical. For a more detailed description of the relationship between the value of Δδ^13^C^α^–Δδ^13^C^β^ and secondary structure of an amino acid residue please see the reference [34].

The effect of the L17A/F19A mutation on the secondary structure of Aβ_40_(E22Q) was also analyzed in terms of the changes of secondary chemical shifts of ^13^C^α^ and ^13^C^β^. Figure 6A,B shows the plots of ^13^C^α^ secondary chemical shifts and the values of Δδ^13^C^α^–Δδ^13^C^β^ of Aβ_40_(E22Q) and Aβ_40_(L17A/F19A/E22Q) as a function of residue, respectively. It can be seen from Figure 6A,B that residues which displayed significant changes in the ^13^C^α^ secondary chemical shifts and the values of Δδ^13^C^α^–Δδ^13^C^β^ as a result of L17A/F19A mutation were mainly located in the α/β-discordant region of Aβ_40_(E22Q). Moreover, both the ^13^Cα secondary chemical shifts and the values of Δδ^13^C^α^–Δδ^13^C^β^ for the residues in the α/β-discordant region are significantly more positive for Aβ_40_(L17A/F19A/E22Q) than for Aβ_40_(E22Q). These findings suggested that Aβ_40_(L17A/F19A/E22Q) adopted two short α-helices from residues 15 to 26 and residues 28 to 34, and residues 15–26 of Aβ_40_(L17A/F19A/E22Q) adopted higher α-helical propensities than those of Aβ_40_(E22Q). It has to be noted that changes of these secondary chemical shifts are primarily contributed by structural conformational changes induced by the L17A/F19A mutation. Alternation of interaction with SDS micelle would result in changes of these secondary chemical shifts as well. We cannot rule out the possibility that interaction of the α/β-discordant region of Aβ_40_(E22Q) with SDS micelle would be altered due to the L17A/F19A mutation. However, whether interaction with SDS is strong or not, its effect on the changes of these secondary chemical shifts is small.

### 2.3. L17A/F19A Mutation Inhibits the Aggregation of Aβ_40_(E22Q)

**Figure 7:**
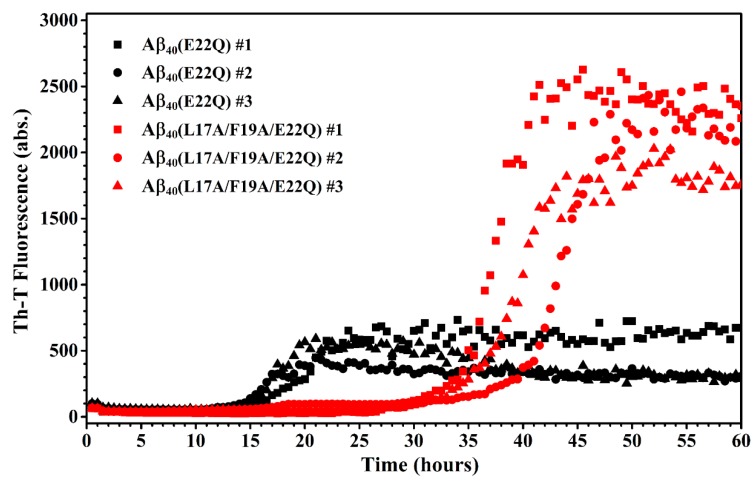
Aggregation kinetics of Aβ_40_(E22Q) (red) and Aβ_40_(L17A/F19A/E22Q) (black).

**Figure 8:**
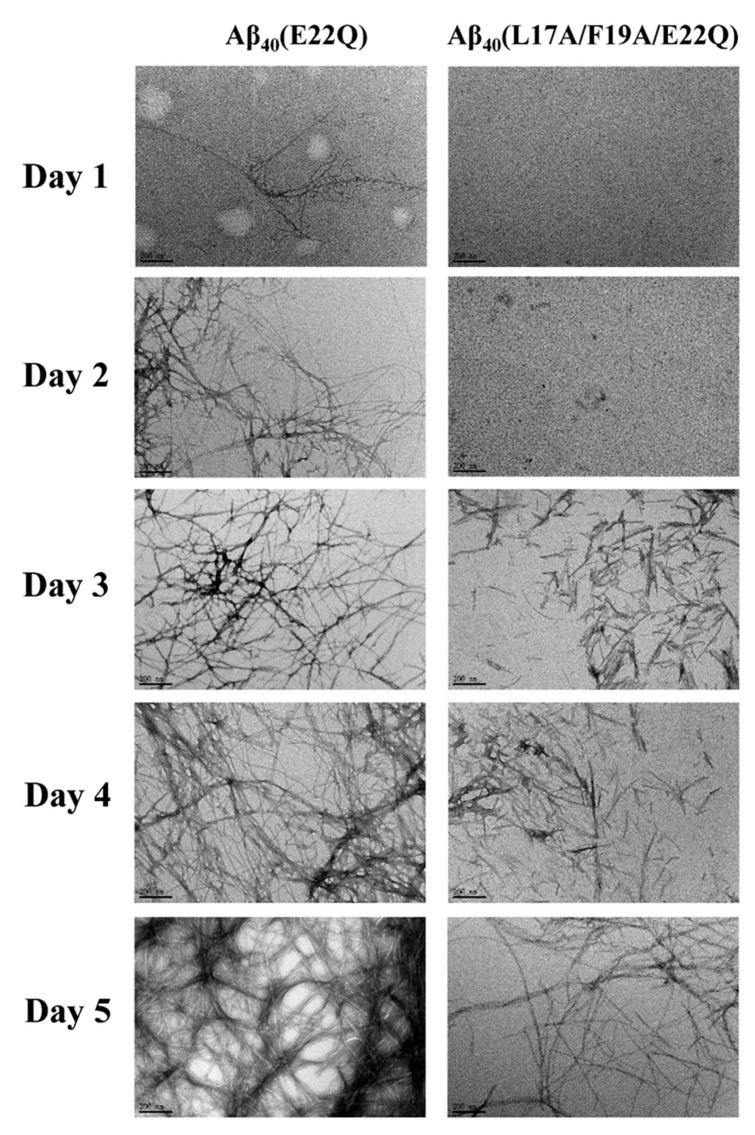
TEM images of Aβ_40_(E22Q) and Aβ_40_(L17A/F19A/E22Q). The scale bar is 200 nm.

We characterized the effect of L17A/F19A mutation on the structural propensity of Aβ_40_(E22Q). However, the effect of L17A/F19A mutation on the aggregative property of Aβ_40_(E22Q) remained unclear. To investigate this issue, we applied thioflavin-T (Th-T) fluorescence assay and transmission electron microscopy (TEM) to monitor the aggregation processes of Aβ_40_(E22Q) and Aβ_40_(L17A/F19A/E22Q) in aqueous solution. The results of Th-T assay and TEM are shown in Figure 7 and Figure 8, respectively. It can be seen from Figure 7 that the shapes of the aggregation profiles of Aβ_40_(E22Q) and Aβ_40_(L17A/F19A/E22Q) in aqueous solution are sigmoidal, suggesting that both peptides aggregated in a nucleation-dependent polymerization manner. Furthermore, the two aggregation profiles shown in Figure 7 displayed two distinct lag phases (nucleation phases) whose durations are 12 and 27 h for Aβ_40_(E22Q) and Aβ_40_(L17A/F19A/E22Q), respectively. This result revealed that Aβ_40_(E22Q) aggregated more rapidly than Aβ_40_(L17A/F19A/E22Q). Figure 8 shows the TEM images of Aβ_40_(E22Q) and Aβ_40_(L17A/F19A/E22Q) in aqueous solution acquired at different time points. Fibrils were observed at Day 1 and Day3 for Aβ_40_(E22Q) and Aβ_40_(L17A/F19A/E22Q), respectively. This observation indicated that the rate of fibril formation is more rapid for Aβ_40_(E22Q) than for Aβ_40_(L17A/F19A/E22Q). Taken together, these findings suggested that the L17A/F19A mutation would reduce the aggregation rate of Aβ_40_(E22Q). From a kinetic point of view, the free energy of activation for conformational change from α-helix to β-strand would be higher for a peptide which adopts a higher α-helical propensity. Since the conformational change from the α-helix to the β-strand of Aβ is one of the key factors in governing its aggregative propensity, it is reasonable to infer that L17A/F19A mutation inhibits the aggregation of Aβ_40_(E22Q). This might be through increasing the α-helical propensity of its α/β-discordant region, which in turn reduces its rate of conformational change from the α-helix to the β-strand.

## 3. Discussion

Recently, Hatami et al. reported the effects of FAD-related mutations within the Aβ sequence on the fibrils morphology and aggregation kinetics of Aβ using TEM and Th-T assay [11]. They found that most FAD-related Aβ mutants exhibited faster rates of aggregation. They also observed that Th-T fluorescence profiles of these FAD-related Aβ mutants displayed shorter times of lag phase with higher intensities of Th-T fluorescence and higher amounts of fibrils as compared to wild-type Aβ_40_, however, not all FAD-related Aβ mutants displayed the same patterns. Several FAD-related Aβ mutants showed a lower intensity of Th-T fluorescence with a higher amount of fibrils. This phenomenon can be explained by the binding ability of Th-T with Aβ aggregates or fibrils, since Th-T would bind to aggregates or fibrils of different structural conformations with distinct binding abilities, resulting in different fluorescence intensities. Hatami et al. reported that the intensity of Th-T fluorescence is not correlated with the amyloid fibril content. It can also be applied to explain our data shown in Figure 7 and Figure 8 in which the Th-T fluorescence intensity of Aβ_40_(L17A/F19A/E22Q) after 45 h was higher than that of Aβ_40_(E22Q) and the TEM images showed a smaller amount of aggregates and/or fibrils of Aβ_40_(L17A/F19A/E22Q). These observations also suggested that the fibril conformation of Aβ_40_(L17A/F19A/E22Q) should be different from that of Aβ_40_(E22Q).

Many studies have reported that the FAD-related mutations, Dutch-type and Arctic-type mutations, both of which are located at position 22 within the Aβ sequence, would result in an increase of the aggregation rate of Aβ [11,12,37,38]. However, the underlying mechanisms by which these two FAD-related mutations accelerate the aggregation process of Aβ remain elusive. In general, the aggregation process of Aβ would involve conformational changes and self-association which are closely related to the intrinsic structural propensity, the intramolecular interactions within the Aβ molecule, and intermolecular interactions between Aβ molecules. Thus, any factor which varies these properties would alter its aggregation behavior as we discussed in the previous paper [30,31]. In the previous study, we investigated the mechanism of why Arctic-type mutation accelerates Aβ aggregation from a structural point of view and proposed that Arctic-type mutation would reduce the α-helical propensity of the α/β-discordant region of Aβ, resulting in an acceleration of Aβ aggregation [30]. However, it remains unclear whether or not Arctic-type mutation would enhance or reduce the intramolecular and/or intermolecular interactions of Aβ, since it is difficult to measure these interactions. In this study, we applied the same approach to investigate the underlying mechanism of how Dutch-type mutation promotes Aβ aggregation. Our data indicated that Dutch-type mutation, unlike Arctic-type mutation, has no significance on the structural propensity of Aβ. According to our data, the structural propensity of Dutch-type Aβ_40_ and its interaction with SDS micelle are almost the same as those of wild-type Aβ_40_. Thus, we speculated that Dutch-type mutation might alter the intramolecular and/or intermolecular interactions of Aβ, leading to an increase of the aggregation rate of Aβ. This is a very likely inference, even though these effects were not directly observed. A single mutation at the same position (position 22) within the Aβ sequence with a different amino-acid would result in a distinct mechanism by which it promotes Aβ aggregation. This might be the reason why different FAD-related mutations within the Aβ sequence displayed different clinical characteristics, such as cerebral amyloid angiopathy (CAA) for Dutch-type Aβ_40_.

For L17A/F19A mutation, our data suggested that one of the factors in determining its inhibition of the aggregation of Aβ_40_(E22Q) is through increasing the α-helical propensity of the α/β-discordant region of Aβ_40_(E22Q). This effect was also observed on wild-type Aβ_40_ and Aβ_40_(E22G) [21,29]. Whether the L17A/F19A mutation could inhibit the aggregation of other FAD-related Aβ mutants through the same effect which is exerted on wild-type Aβ_40_, Aβ_40_(E22Q) and Aβ_40_(E22G) remains to be investigated. The possibility that the intramolecular and/or intermolecular interactions of Aβ would be altered by the L17A/F19A mutation cannot be ruled out. We characterized the effects of L17A/F19A mutation on the structural propensity and aggregation kinetics of wild-type, Arctic-type and Dutch-type Aβ_40_, however, the effects of L17A/F19A mutation on the structural propensity and aggregation kinetics of the more amyloidogenic Aβ_42_ and its FAD-related mutants remain unclear. Since the fibril structures of Aβ_40_ [25,26,28,39] and Aβ_42_ [40,41,42,43,44,45] have been solved at the atomic resolution, the intramolecular interactions within Aβ molecule and intermolecular interactions between Aβ molecules can be grasped to some extent based on this structural information. The intramolecular interactions within Aβ_40_ were located at K16-D23 and G29-M35 segments, which correspond to the α/β-discordant region and c-terminal α-helix, respectively. According this structural information, L17A/F19A mutation would disrupt the intramolecular interaction within Aβ_40_. It can be seen from the fibril structure of Aβ_42_ that residues Ile41 and Ala42 are involved in the intramolecular and intermolecular interactions, suggesting that these two residues would affect the aggregation kinetics of Aβ. According to the Aβ_42_ fibril structure, we may also speculate that the L17A/F19A mutation would also disrupt the intramolecular interaction within Aβ_42_, leading to an alternation of the aggregative propensity of Aβ_42_. Knowing the effects of the mutations within the Aβ sequence may help us in developing agents for inhibition of the aggregation of Aβ.

## 4. Materials and Methods

### 4.1. Preparation of Aβ Peptides

The protocols for production of Aβ peptides were the same as those described in the previous studies [29,30,31]. All peptide samples were dissolved in 70% TFE (trifluoroethanol) and then lyophilized. For NMR studies, peptides were dissolved in 0.25 mL 100 mM SDS-d_25_ (sodium dodecyl sulfate-d_25_) with 10% (*v*/*v*) D_2_O/H_2_O containing 10 mM phosphate buffer at pH 6.0. TSP (3-(trimethylsilyl)propionic-2,2,3,3,-d_4_ acid) was used for internal chemical shift standard. The sample solutions were put into the 5 mm Shigemi tubes (Shigemi Co., Allison Park, PA, USA) for NMR spectra recording.

### 4.2. Nuclear Magnetic Resonance (NMR) Spectroscopy

NMR experiments were performed at 296 K on the Bruker AVANCE-500, 600, or 800 spectrometer equipped with a 5-mm inverse triple resonance (^1^H/^13^C/^15^N), Z-axis gradient cyroprobe. NMR data were processed and analyzed using the TopSpin and AURELIA programs (Bruker BioSpin GmbH, Rheinstetten, Germany). Linear predictions were used in the indirectly detected dimensions to improve digital resolution. ^1^H chemical shifts were referenced to the ^1^H frequency of the methyl resonances of TSP at 0 ppm. The ^15^N and ^13^C chemical shifts were indirectly referenced using the following consensus ratios of the zero-point frequencies: 0.101329118 for ^15^N/^1^H and 0.251449530 for ^13^C/^1^H [46]. Backbone sequential assignments were accomplished using the three-dimensional spectra: HNCOCA, HNCO, HNCA, and CBCA(CO)NH [30,31].

### 4.3. Circular Dichroism (CD) Spectroscopy

Pretreated Aβ peptides (50 μM) were dissolved in 0.160 mL 100 mM SDS containing 5 mM phosphate buffer at pH 6.0. CD measurements were performed on an AVIV CD spectrometer (Aviv 410 spectropolarimeter, Aviv Biomedical, Inc., Lakewood, NJ USA) at 296 K [30,31]. The measurement was carried out three times.

### 4.4. Thioflavin T (Th-T) Fluorescence Assay

Pretreated Aβ peptides (30 μM) were incubated in aqueous solution (5 mM phosphate buffer, pH 7.2). The molar ratio of Aβ and thioflovin T (Th-T) (Sigma) was 1:1. Fluorescence signals were acquired (SpectraMax M5, Molecular Device, San Jose, CA, USA) every 30 min at 37 °C. The excitation and emission wavelengths of fluorescence were 450 nm and 482 nm, respectively [21,29].

### 4.5. Transmission Electron Microscopy (TEM)

TEM images of Aβ peptides were acquired using JEOL JEM-2100 EXII TEM (JEOL, Tokyo, Japan). Pretreated Aβ peptides (60 μM) were dissolved in 10 mM phosphate buffer, pH 7.0 and incubated at 37 °C for different time periods. Sample preparation of TEM followed the procedures as described [29,30].

## 5. Conclusions

In the present study, we characterized the effects of E22Q and L17A/F19A mutations on the structural propensities of wild-type Aβ_40_ and Aβ_40_(E22Q), respectively, by CD and NMR spectroscopy. We found that the E22Q mutation has no significant effect on the structural propensity of wild-type Aβ_40_, indicating that it does not promote aggregation by altering the α-helical propensity of the α/β-discordant region. This finding supported the view that it is not necessary for FAD-related mutations in the α/β-discordant region to promote aggregation by altering the structural propensity of Aβ. Besides wild-type and Arctic-type Aβ_40_, the L17A/F19A mutation would increase the α-helical propensity of the α/β-discordant region of Dutch-type Aβ_40_, resulting in inhibition of Aβ_40_(E22Q) aggregation. It is possible that the L17A/F19A mutation can be applied to inhibit Aβ aggregation in vivo. This study provides the information for a clearer understanding of how mutations within the α/β-discordant region of Aβ affect aggregation.

## Abbreviations

Aβ	β-amyloid peptide
βAPP	β-amyloid precursor protein
AD	Alzheimer’s disease
FAD	familial Alzheimer’s disease
CAA	cerebral amyloid angiopathy
NMR	nuclear magnetic resonance
CD	circular dichroism
TEM	transmission electron microscopy
SDS	sodium dodecyl sulfate
TFE	trifluoroethanol
Th-T	thioflavin-T
